# Change your Angle of View: Sinusoidal C-Arm Movement in Cranial Flat-panel CT to Improve Image Quality

**DOI:** 10.1007/s00062-022-01172-z

**Published:** 2022-05-05

**Authors:** Hannes Luecking, Philip Hoelter, Stefan Lang, Manuel Schmidt, Felix Eisenhut, Arnd Doerfler

**Affiliations:** Neuroradiological Institute, University of Erlangen-Nuremberg, Schwabachanlage 6, 91054 Erlangen, Germany

**Keywords:** Neurointervention, Posterior fossa, Dyna CT, Sine spin, Artifact reduction

## Abstract

**Background:**

Artifacts from surrounding bony structures, especially from the petrous bones, regularly impair soft tissue computed tomography (CT) imaging of the middle and posterior fossa. This affects flat-panel CT in particular. Sinusoidal movement of the C‑arm during acquisition (i.e. craniocaudal tilting along with semicircular rotation) is supposed to reduce artifacts, thus enhancing soft tissue imaging quality.

**Methods:**

In the work-up of ischemic stroke or subarachnoid hemorrhage 40 patients underwent multi-slice CT (MS-CT) and either plain circular (cFP-CT; *n* = 20) or sinusoidal (sFP-CT; *n* = 20) flat-panel CT within a short interval. Two independent readers analyzed MS-CT and FP-CT datasets for recognizability of eight different brain structures and three typical types of artifacts according to a predetermined score.

**Results:**

Interrater reliability was moderate for cFP-CT (κ = 0.575) and good to very good for ratings of MS-CT and sFP-CT (κ = 0.651 to κ = 1). MS-CT was rated to be significantly better than cFP-CT and sFP-CT (*p* < 0.0001) in the overall score. Yet, sFP-CT was rated to be significantly superior to cFP-CT (overall *p* < 0.0001) regarding most anatomical regions and petrous bone artifacts.

**Conclusion:**

Compared to a standard circular protocol, sinusoidal C‑arm movement in cranial FP-CT can significantly reduce artifacts in the posterior fossa and, moreover, can improve visualization of most supratentorial and infratentorial anatomical structures.

**Supplementary Information:**

The online version of this article (10.1007/s00062-022-01172-z) contains supplementary material, which is available to authorized users.

## Introduction

Flat-panel computed tomography (FP-CT) has been available for many years in the surrounding of cerebral digital subtraction angiography (DSA), e.g. for therapy planning [1, 2]. Contrast-enhanced 3D datasets can be beneficial for the work-up of several kinds of pathologies from simple cerebral aneurysms to complex arteriovenous malformations.

Non-enhanced FP-CT can be used to replace multi-slice cranial CT (MS-CT) in certain settings. It allows for MS-CT-like imaging without having to move patients to the CT suite, thus reducing transportation time. Typical settings could involve re-evaluation of patients with acute stroke after relocation to a thrombectomy center or verification of proper placement of ventricular drainages in patients with subarachnoid or intraventricular hemorrhage prior to DSA (one-stop-shopping).

Although image quality has improved for both modalities over the years [3], FP-CT performs still behind MS-CT regarding soft tissue contrast and bony hardening artifacts. This affects the posterior fossa in particular, especially the brainstem.

**Fig. 1:**
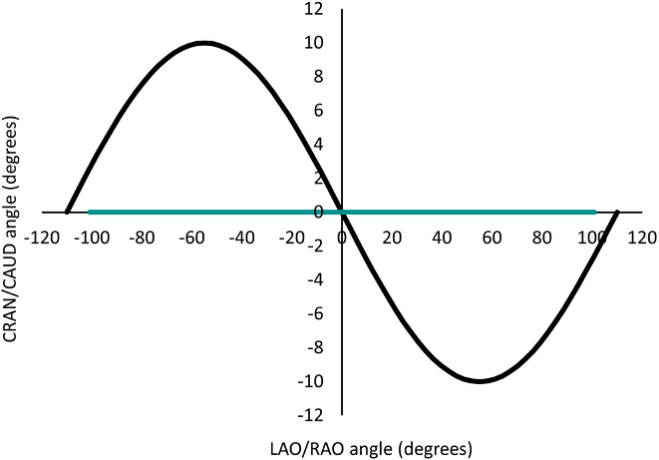
Trajectories of circular flat-panel CT (cFP-CT, *teal curve*) and sinusoidal flat-panel CT (sFP-CT, *black curve*). In sFP-CT, an additional component of 10 ° of cranial and caudal movement is added to the circular path (image courtesy of Siemens Healthineers)

In hitherto FP-CT the C‑arm follows a plain (semi)circular path. Sinusoidal movement adds a craniocaudal component of motion along this circular path that allows for varying angels of view to the volume of interest. Fig. 1 illustrates the trajectory of the C‑arm along its circular and craniocaudal path. This is supposed to reduce the impact of hardening artifacts from adjacent bony structures, thus improving soft tissue imaging quality.

This evaluation compares MS-CT to FP-CT runs of the respective patients, 2 groups of each 20 patients (40 patients in total) underwent either MS-CT and circular FP-CT (cFP-CT) or MS-CT and sinusoidal FP-CT (sFP-CT).

## Methods

### Inclusion

From our database a total of 40 patients were selected who had undergone cFP-CT and MS-CT (*n* = 20) or sFP-CT and MS-CT (*n* = 20) within 3 h prior to FP-CT. Whereas MS-CT is done as first line imaging for most patients arriving via the emergency ward, FP-CT is performed regularly in our center in stroke and SAH work-up to rule out procedure-related complications after neuroendovascular interventions. Datasets with major foreign objects, e.g. large coil packages, implanted in between MS-CT and FP-CT, were excluded in order to obtain good comparability of soft tissue contrast. Additional comparison of FP-CT to MS-CT as well as comparison of both MS-CT subgroups among each other were chosen to ensure general comparability of both FP-CT subgroups (rule-out of casual bias in FP-CT subgroup allocation).

SFP-CT is set as system standard in our routine setup of the angiographic system. For collection of cFP-CT datasets, the system standard was changed from sFP-CT to cFP-CT for a time span of 3 months.

### CT-Imaging

MS-CT was done on a 128-row multi-slice scanner Somatom Definitions AS+ (Siemens Healthineers, Forchheim, Germany). Secondary reconstructions were drawn for the analysis with 4.8 mm slice thickness and 4.8 mm spacing. Predetermined center value was 40 HU and window width was 100 HU. Changing center and window settings was allowed during assessment.

### FP-CT Imaging

Native 8s circular FP-CT and 7s sinusoidal FP-CT runs were performed on an ICONO biplane angiographic system (Siemens Healthineers, Forchheim, Germany), with both protocols being factory standard. In analogy to the MS-CT reconstruction protocol, 4.8 mm reconstructions were done with manual setting of center and window values. Again, changing center and window settings during assessment was allowed.

SFP-CT acquires 100 more projections than cFP-CT (596 vs. 496). This results in a coverage of 220° in sFP-CT rather than 200° in cFP-CT. Furthermore, sFP-CT uses 4 × 4 binning instead of 2 × 2 binning in cFP-CT. Binning describes the summation of the signal of several detector units in electronic post-processing, with binning of more units resulting in a smoother image. For further scan parameters of MS-CT and FP-CT see Table 1.

**Table 1:** Scan parameters for circular and sinusoidal protocols

	Voltage	Angle	Stepping	Projections	Binning^a^	Dose
8s circular	109 kV	200°	0.04°/frame	496	2 × 2	1.820 μGy/frame
7s sinusoidal	109 kV	220°	0.04°/frame	596	4 × 4	1.820 μGy/frame

^a^ Pooling of several detector elements (higher binning results in lower spatial resolution and lower noise and vice versa)

### Imaging Evaluation and Statistical Analysis

All 80 datasets were evaluated independently by two readers with more than 10 years of experience in neuroimaging. A simple scoring system was created to comprehensively assess brain soft tissue, bony and foreign object hardening artifacts. Sinusoidal C‑arm movement is mainly supposed to reduce petrous bone artifacts in the posterior fossa. Besides assessing the brainstem and cerebellum, also supratentorial structures were included in the scoring system in order to detect whether the technique could also provide advantages in other anatomical regions. This led to a catalogue of eight anatomic structures and another three types of artifacts being assessed, each with a threefold selection, resulting in a maximum score of 33 points (Table 2).

**Table 2:** Assessment results for both raters (mean value through all patients) using Mann-Whitney-U-Test. Last column gives significance level for comparison between 8s circular and 7s sinusoidal runs

	Rater 1 & 2				
Criterion	MS-CT	8s circular	MS-CT	7s sine spin	*p* value 8s/7s
GWM^a^ frontal^b^	3	2.6	3	2.8	*p* = 0.042
GWM^a^ parietal^b^	3	2.4	3	2.7	*p* = 0.012
GWM^a^ temporal^b^	3	1.8	2.9	1.9	*p* = 0.164
GWM^a^ occipital^b^	3	1.7	3	2.1	*p* = 0.012
Insular cortex^b^	2.9	1.8	3	2	*p* = 0.142
Basal ganglia^b^	2.9	2.1	3	2.5	*p* = 0.003
Brainstem^b^	2.5	1.2	2.6	1.8	*p* < 0.0001
Cerebellum^b^	3	1.2	3	1.5	*p* = 0.002
Petrous bone artifacts^c^	2.7	1.4	3	1.9	*p* < 0.0001
Skull hardening artifacts^c^	3	2.3	3	2.4	*p* = 0.169
Foreign objects artifacts^c^	3	3	3	2.9	*p* = 0.352
Sum	31.8	21.2	32.3	24.4	*P* < 0.0001

^a^ Grey/white matter contrast

^b^ Good (3 points), fair (2 point), poor (1 point)

^c^ Mild (3 points), mediocre (2 points), severe (1 point)

Also, the recognizability of pathologies correlated with the actual cause of admission was recorded but was not part of the scoring system. Regarding tissue assessment there were 3 grades (good: 3 points; fair: 2 points; poor: 1 point) while for artifact assessment the grading was counted contrariwise (no/minor artifacts: 3 points; 2 mediocre artifacts: 2 points; severe artifacts: 1 point).

**Fig. 2:**
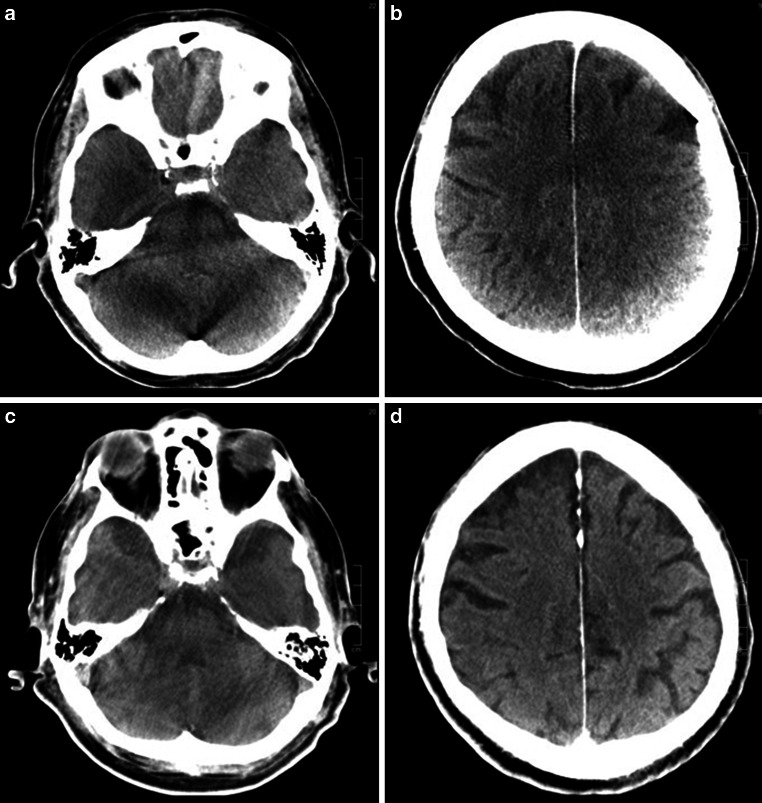
*Upper row* (**a**/**b**) shows infratentorial and frontoparietal slices taken from circular FP-CT. Note marked artifacts from the skull base, petrous bones and internal occipital protuberance (**a**) and inhomogeneous contrast especially in the parietal lobes (**b**). *Lower row* is taken from sinusoidal FP-CT of a different person. Note reduced posterior fossa artifacts with better visualization of brainstem and 4th ventricle (**c**) and more homogeneous contrast of the cerebrum with improved differentiation of grey and white matter (**d**)

Blinding the readers to the type of imaging (MS-CT vs. FP-CT) was not appropriate as the nature of the respective reconstruction is obvious to an experienced reader (see Fig. 2).

SPSS 28 (IBM, Armonk, NY, USA) was used for statistical analysis. Ordinal values were checked for normal distribution using Kolmogorov-Smirnov test. Due to the mostly non-normal distribution, Mann-Whitney U‑test was used to compare subgroups. Inter-rater correlation was measured using Cohen’s kappa κ.

## Results

Patients were admitted for either ischemic stroke (*n* = 16 for the cFP-CT group; *n* = 18 for the sFP-CT group) or for subarachnoid hemorrhage (*n* = 4 vs. *n* = 2) and, thus underwent MS-CT immediately after admission and FP-CT in the course of thrombectomy or SAH-/aneurysm work-up.

FP-CT was consistently performed with the patient under general anesthesia in the treatment setting of their respective primary disease in all patients, ruling out motion artifacts.

### Assessment Rating

Both MS-CT groups (circular control and sinusoidal control) were not found to differ significantly regarding overall assessment score by both readers (*p* = 0.100; 95% CI), indicating that both the circular and sinusoidal groups had no major bias (e.g. foreign material) systematically compromising multi-slice and flat-panel imaging. Table 2 provides combined results of both readers for all assessed anatomical structures and artifacts. Separate results of both raters are provided in supplementary table S2. SFP-CT was rated significantly superior to cFP-CT in assessment of grey/white matter contrast of the frontal, parietal and occipital lobes, and visualization of basal ganglia, brainstem and cerebellum. Petrous bone artifacts were found to be significantly less in sFP-CT runs. No significant difference was found for the temporal lobe and insular cortex and skull and foreign objects hardening artifacts.

Both sFP-CT (mean score = 24.5; *p* < 0.0001) and cFP-CT (mean score = 21.3; *p* < 0.0001) runs were found to perform significantly lower than MS-CT (mean score = 32.0) by both raters with respect to the overall score.

### Interrater Reliability

Cohen’s kappa was calculated to check interrater reliability in assessment of visibility of anatomical structures and artifacts. Each subset (cFP-CT, sFP-CT, circular control and sinusoidal control) was calculated separately. Results are provided in Table 3. According to the interpretation of Altman, interrater reliability was good (κ ≥ 0.60) or very good (κ ≥ 0.80) regarding assessment of anatomical structures and artifacts in all subgroups, except for anatomical structures in cFP-CT, where reliability was found to be moderate (κ ≥ 0.40).

**Table 3:** Interrater (IR) reliability for assessment of anatomical structures and artifacts for separate modalities

	IR reliability anatomy	IR reliability artifacts
8s multi-slice control	κ = 0.651	κ = 0.621
8s circular FP-CT	κ = 0.575	κ = 0.821
7s multi-slice control	κ = 0.656	κ = 1
7s sinusoidal FP-CT	κ = 0.842	κ = 0.757

### Admission-Related Pathology

**Fig. 3:**
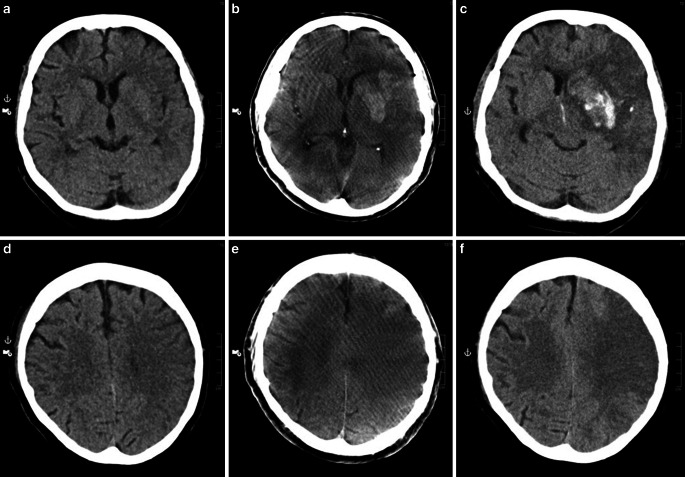
Imaging over time in a case of acute carotid‑T occlusion. **a/d** show qualifying image (multi-slice CT) for endovascular therapy with preserved contrast of basal ganglia (**a**) and frontal and parietal lobes (**d**). **b/e** show contrast extravasation in basal ganglia (**b**) and beginning demarcation in the frontal and parietal lobes (**e**), (sinusoidal flat-panal CT after endovascular therapy). **c/f** show 24 h follow-up (multi-slice CT) with progressive demarcation of the basal ganglia (**c**) and more apical levels (**f**)

Both raters found a higher number of pathologic findings in the cFP-CT and sFP-CT datasets compared to their respective MS-CT groups (rater 1: MS-CT:cFP-CT=15:19; MS-CT:sFP-CT=11:22; rater 2: MS-CT:cFP-CT=16:17; MS-CT:sFP-CT=11:22). This demonstrates the general ability of FP-CT of either kind to detect and assess admission-related pathologies. Yet, given the time interval of mean 146 ± 30.2 min (range 63–189 min) between MS-CT and FP-CT, it lies in the nature especially of ischemic stroke to show progressive visibility in the course of time (Fig. 3).

### Radiation Dose

The dose area product (DAP) was significantly higher in sFP-CT (mean 5076 µGym^2^) than in cFP-CT (mean 4583 µGym^2^), *p* = 0.0021.

## Discussion

Non-enhanced flat panel CT of the brain as an alternative for “conventional” multi-slice CT can be used for multiple purposes in the surrounding of neuroendovascular imaging and treatment. Imaging quality, yet, is inferior to MS-CT regarding soft tissue contrast and susceptibility to bone hardening artifacts, which affects the posterior fossa in particular. Sinusoidal C‑arm movement is supposed to reduce petrous bone and hardening artifacts in general, by providing different angles of view not only along a circular path but also in the craniocaudal direction.

It was the aim of this analysis to compare image quality of cFP-CT and sFP-CT among each other and to common MS-CT.

### Imaging Characteristics

For reading of MS-CT, a standard center/window setting of 40/100 Hounsfield units is used, which is suitable in most cases. FP-CT, on the other hand, was found to require distinctive changes to center and window settings to assess different areas, e.g. cerebral cortex, basal ganglia or middle and posterior fossa, in all datasets. This increases assessment effort and could, depending on the reader’s experience and dedication, lead to a more reader-dependent quality of reporting in FP-CT.

With less artifacts especially in the posterior fossa, effort to find appropriate window settings is, by subjective perception, slightly reduced in the sFP-CT group (see Fig. 2, row 2), but the basic statement remains true for both groups of FP-CT.

SFP-CT is reconstructed with a 4 × 4 binning of detector elements, as opposed to 2 × 2 binning in cFP-CT. As there are no factory standard protocols available that match regarding this setting, it cannot be excluded that the higher binning in sFD-CT could, by reduction of noise, also contribute to the more favorable results of sFP-CT. Setting up a respective sFP-CT protocol with 2 × 2 binning required major changes to the system including recalibration and consistency check and, thus, was not meaningful for the evaluation.

### Interrater Reliability

Although both raters have long experience in assessing FP-CT and interrater reliability showed mostly “good” and “very good” reliability, there was only “moderate” reliability for cFP-CT. This indicates that the individual and subjective perception of imaging quality may differ relevantly, especially if the quality is below gold standard (MS-CT). Pathological findings of clinical relevance, yet, can be depicted reliably by FP-CT, as shown by other authors [4]. Interrater reliability regarding assessment of anatomical structures was best for sFP-CT. This raises the assumption that improvement of imaging characteristics and quality as provided with sFP-CT can contribute to making reading of FP-CT more reliable, potentially enabling readers to gather information more easily.

### Assessment Results

With an average of 32/33 points (97%) MS-CT was rated significantly better regarding assessment of anatomical structures and susceptibility to artifacts compared to sFP-CT (24.5/33 points, 74%) and cFP-CT (21.3/33 points, 65%), (*p* < 0.0001, 95% CI). Yet, sFP-CT was found to be significantly better than cFP-CT (*p* < 0.0001) in the overall score and in most anatomical areas except the temporal lobe and insular cortex. Gray/white matter contrast in the frontal, occipital and parietal lobes was visualized significantly better (Fig. 2, right column), possibly allowing the visual or automated determination of aspects scores in one-stop-shopping or post thrombectomy scans [5–7]. Petrous bone artifacts, which are one key point when it comes to image quality in the posterior fossa, were rated to be significantly lower in sFP-CT (*p* < 0.0001), going along with a, again, significantly better visualization of the brainstem and cerebellum.

Although visualization of the temporal lobe and insula was rated slightly better in sFP-CT than in cFP-CT, the result was not significant. The authors assume that this is caused by scatter radiation from the skull base and the lower soft tissue resolution of FP-CT, which affects cFP-CT and sFP-CT similarly.

If admission-related pathologies were discovered, this was also noted. Due to the natural course of ischemic brain lesions, FP-CT revealed more pathologies than MS-CT, which consistently was performed prior to FP-CT in the course of diagnostics and treatment. Although not assessed in particular, this indicates that FP-CT—regardless of scan parameters—can be able to detect relevant pathologies, such as hemorrhage or subacute infarcts.

### Radiation Dose

The dose-area product (DAP) that is automatically calculated and displayed by the angiographic system, was approximately 10.7% (mean ~500 µGym^2^) higher in sFP-CT than in cFP-CT. Sinusoidal scans do not allow for craniocaudal collimation of the beam to make sure that the whole volume of interest is covered along the trajectory of the C‑arm. Furthermore, a coverage of 220° in sFP-CT rather than 200° in cFP-CT adds to an increase in DAP. The lack of possible collimation may lead to the assumption that a relevant share of the additional radiation probably does not interfere with the patient. Yet, these peculiarities of sFP-CT should be kept in mind as, for instance, eye lenses cannot to be spared by proper bedding of the patient’s head [8–10].

### Limitations

The study is limited by the retrospective design and limited number of patients. Comparison of effective radiation dose would require elaborate phantom tests.

## Conclusion

Sinusoidal C‑arm movement in flat-panel imaging as new feature of recent angiographic systems provides significantly superior image quality of supratentorial and infratentorial brain tissue compared to regular “circular” flat-panel CT. Petrous bone artifacts are significantly reduced.

## Supplementary Information

Table S2: Results of assessment per rater (mean value through all patients). Last column gives significance level for comparison between 8s circular and 7s sine-spin runs. *) Grey/white matter contrast; 1) good (3 points), fair (2 point), poor (1 point); 2) mild (3 points), mediocre (2 points), severe (1 point)
