# Supplementary material for: Change your Angle of View: Sinusoidal C-Arm Movement in Cranial Flat-panel CT to Improve Image Quality
Source: Clin Neuroradiol. 2022 May 5;32(4):1109–15. doi: 10.1007/s00062-022-01172-z (PMC9744702; doi:10.1007/s00062-022-01172-z)
Supplement: Supplementary file 1 — Table S2: Results of assessment per rater (mean value through all patients). Last column gives significance level for comparison between 8s circular and 7s sine-spin runs. *) Grey/white matter contrast; 1) good (3 points), fair (2 point), poor (1 point); 2) mild (3 points), mediocre (2 points), severe (1 point) [file 62_2022_1172_MOESM1_ESM.docx]

Table S2: Results of assessment per rater (mean value through all patients). Last column gives significance level for comparison between 8s circular and 7s sine-spin runs. *) Grey/white matter contrast; 1) good (3 points), fair (2 point), poor (1 point); 2) mild (3 points), mediocre (2 points), severe (1 point)

|  | Rater 1 | | | | |
| --- | --- | --- | --- | --- | --- |
| Criterion | MS-CT | 8s circular | MS-CT | 7s sine spin | p value 8s/7s |
| GWM* frontal^1^ | 3 | 2.7 | 3 | 2.8 | p=0.48 |
| GWM* parietal^1^ | 3 | 2.5 | 3 | 2.8 | p=0.08 |
| GWM* temporal^1^ | 3 | 1.7 | 2.9 | 1.9 | p=0.23 |
| GWM* occipital^1^ | 3 | 1.6 | 3 | 2.1 | p=0.009 |
| Insular cortex^1^ | 2.9 | 1.8 | 3 | 2 | p=0.25 |
| Basal ganglia^1^ | 2.9 | 2.3 | 3 | 2.6 | p=0.08 |
| Brainstem^1^ | 2.5 | 1.2 | 2.6 | 1.8 | p<0.0001 |
| Cerebellum^1^ | 3 | 1.2 | 3 | 1.6 | p=0.02 |
| Petrous bone artifacts^2^ | 2.7 | 1.4 | 3 | 2 | p<0.0001 |
| Skull hardening artifacts^2^ | 3 | 2.4 | 3 | 2.6 | p=0.25 |
| Foreign objects artifacts^2^ | 3 | 3 | 3 | 2.9 | p=0.16 |
| Sum | 31.8 | 21.8 | 32.3 | 25 | p=0.001 |
|  |  |  |  |  |  |
|  | Rater 2 | | | | |
| Criterion | MS-CT | 8s circular | MS-CT | 7s sine spin | p value 8s/7s |
| GWM* frontal^1^ | 3 | 2.4 | 3 | 2.8 | p=0.013 |
| GWM* parietal^1^ | 3 | 2.4 | 3 | 2.6 | p=0.08 |
| GWM* temporal^1^ | 3 | 1.8 | 3 | 1.9 | p=0.28 |
| GWM* occipital^1^ | 3 | 1.8 | 3 | 2 | p=0.13 |
| Insular cortex^1^ | 2.9 | 1.9 | 3 | 2 | p=0.27 |
| Basal ganglia^1^ | 3 | 2 | 3 | 2.5 | p=0.001 |
| Brainstem^1^ | 2.6 | 1.2 | 2.6 | 1.8 | p<0.0001 |
| Cerebellum^1^ | 2.8 | 1.1 | 2.9 | 1.5 | p=0.002 |
| Petrous bone artifacts^2^ | 2.8 | 1.4 | 3 | 1.9 | p=0.0004 |
| Skull hardening artifacts^2^ | 2.9 | 2.1 | 3 | 2.2 | p=0.31 |
| Foreign objects artifacts^2^ | 3 | 2.9 | 3 | 2.9 | p=0.5 |
| Sum | 31.7 | 20.9 | 32.3 | 24 | p=0.001 |
